# Effects of Different Diets on Growth Performance and Nutritional Composition of Blue Crab (*Callinectes sapidus,* Rathbun, 1896) in a Recirculating Aquaculture System

**DOI:** 10.3390/ani15192794

**Published:** 2025-09-25

**Authors:** Deniz Devrim Tosun, Güneş Yamaner, Mustafa Yıldız, Şehnaz Yasemin Tosun, Menekşe Didem Demircan Akyasan, Ömer Metin, Eylül Balcıoğlu

**Affiliations:** 1 Aquaculture Division, Department of Aquaculture and Fish Diseases, Faculty of Aquatic Sciences, Istanbul University, 34093 Istanbul, Türkiye; gyamaner@istanbul.edu.tr (G.Y.); mstar@istanbul.edu.tr (M.Y.); omer.metin@istanbul.edu.tr (Ö.M.); 2 Seafood Processing Technology Division, Department of Fisheries and Seafood Processing Technology, Faculty of Aquatic Sciences, Istanbul University, 34093 Istanbul, Türkiye; yasemin@istanbul.edu.tr; 3 Fish Diseases Division, Department of Aquaculture and Fish Diseases, Faculty of Aquatic Sciences, Istanbul University, 34093 Istanbul, Türkiye; dican@istanbul.edu.tr (M.D.D.A.); eylulbalcioglu@ogr.iu.edu.tr (E.B.)

**Keywords:** blue crab, aquaculture, recirculating systems, *Callinectes sapidus*

## Abstract

This study evaluated the effects of three feeding regimens—commercial feed (CF), minced trash fish (MTF), and a 50:50 mixture (MIX)—on the growth performance and tissue composition of the blue crab (*Callinectes sapidus*) reared in a recirculating aquaculture system (RAS). A total of 96 crabs (8 per tank, four tanks per group) were fed daily at 3% of body weight. Growth was assessed via weight gain, carapace width (CW), and carapace length (CL), while biochemical analyses focused on lipid, fatty acid, and amino acid profiles. The CF and MIX groups exhibited significantly higher final body weights (115.64 ± 3.03 g and 113.53 ± 2.53 g, respectively) and CW (97.88 ± 1.02 mm and 97.16 ± 0.43 mm) compared to the MTF group (*p* < 0.05), suggesting more frequent or complete molting. The MTF group showed limited growth (102.81 ± 2.25 g; 95.24 ± 1.19 mm), likely due to reduced ecdysis events. Tissue lipid and fatty acid profiles reflected dietary inputs, with significant differences in DHA/EPA and n-3/n-6 ratios among treatments (*p* < 0.05). Essential amino acid levels decreased across all groups, possibly due to the metabolic demands of molting. These findings highlight the critical role of diet in promoting molting and improving tissue quality in blue crab aquaculture under controlled RAS conditions.

## Simple Summary

Understanding how diet influences blue crab growth and nutritional value is key to improving aquaculture practices. This study tested three diets—commercial pellets, minced fish, and a 50:50 mixture—under controlled conditions. Crabs fed the pellet or mixed diet grew more and had better tissue nutrient profiles than those fed only fish. The mixed diet showed similar growth to pellets, suggesting potential benefits of combining natural and formulated feeds. These results highlight the importance of feed composition not only for growth but also for product quality. This study contributes to sustainable blue crab farming by identifying feeding strategies that support both animal performance and efficient resource use.

## 1. Introduction

The blue crab (*Callinectes sapidus*) is a commercially and ecologically important species native to the western Atlantic and Gulf of Mexico, playing a key role in estuarine food webs as both predator and prey [1,2]. In recent decades, it has become invasive in the Mediterranean Sea, establishing populations in coastal habitats of Türkiye, Greece, Italy, and beyond [3,4,5,6]. Its spread has caused ecological and economic issues by preying on native species, damaging gear, and competing with valuable crustaceans. In Türkiye, many fishers view it as a nuisance due to the dominance of low-value, hard-shell individuals that often damage nets. However, this abundance has also led to targeted fisheries, presenting both challenges and economic potential. Notably, soft-shell crabs yield higher market prices and have attracted producers to harvest them through controlled molting, offering profitable prospects for fisheries and aquaculture [7,8,9,10].

One of the most promising uses of RAS in blue crab aquaculture is the controlled production of soft-shell crabs [11,12,13]. By closely monitoring molting in a controlled environment, crabs can be harvested before their shells harden, minimizing losses and ensuring high product quality [14,15]. RAS systems also enable ranching of wild-caught crabs by maintaining them under optimal conditions until molting, increasing the value of wild stocks [16]. This method not only boosts economic returns but also helps manage invasive populations, turning an ecological threat into a marketable resource [9]. It supports both environmental and economic sustainability in the Mediterranean and other affected areas [17].

Blue crabs (*Callinectes sapidus*) are opportunistic omnivores with a strong preference for animal-based prey, though they can consume plant matter and detritus [18,19]. Their diet is key to optimizing growth and nutritional quality, requiring high levels of protein, lipids, and essential micronutrients for development, molting, and reproduction [20,21,22]. Cannibalism is common, particularly during vulnerable molting stages in late larvae and juveniles [23,24]. These traits highlight the need for protein-rich, animal-based feeds in captive systems, as they help reduce aggression, support survival, and mimic natural feeding behavior. Feeding strategies in RAS must consider their carnivorous nature, molting cycles, and cannibalism risk to ensure welfare and growth [25].

Balanced feeding regimes rich in essential amino acids, fatty acids, and vitamins are vital for optimal growth and body condition in blue crabs [26]. Omega-3 and omega-6 fatty acids support cell membrane development, immune function, and overall physiology [27]. Deficiencies can impair growth, delay molting, and increase disease risk, lowering production efficiency and product quality [28,29]. In RAS, feed composition and practices also affect water quality. Overfeeding or poorly formulated diets raise nitrogenous waste—especially ammonia—straining biofilters and destabilizing the system [30,31]. Thus, efficient feeding is key to maintaining crab health and water quality while minimizing nitrogen and solid waste buildup.

With growing interest in blue crab aquaculture, research is needed to optimize feeding regimes in recirculating aquaculture systems (RASs) to improve efficiency and nutritional quality. While RASs are widely used for finfish and shrimp, their application to crustaceans like *Callinectes sapidus* is still limited. Studies on species such as *Litopenaeus vannamei* and *Procambarus clarkii* show that RASs can support high-density cultures with effective water quality control [32,33]. However, data on blue crab culture in RASs and diet optimization remain scarce. Although RASs offer controlled conditions that can boost growth and survival, success depends heavily on diets tailored to the species’ nutritional needs [30]. Feed composition and digestibility also influence waste output; high-protein feeds, if poorly metabolized, can elevate total ammonia nitrogen (TAN) and degrade water quality. Research on other crustaceans underscores the role of feed management in reducing nutrient pollution and enhancing system sustainability [33,34]. *Callinectes sapidus* undergoes a hormonally regulated molting cycle, where dietary components like cholesterol and specific fatty acids are essential. Cholesterol peaks in hypodermal membranes post-ecdysis, influencing calcium permeability and cuticle mineralization [35,36]. Additionally, n-3 HUFAs and balanced amino acids are vital for successful molting and growth [19]. These insights highlight the need for diets that support both structural and metabolic requirements in cultured blue crabs.

The effectiveness of different diets, whether formulated diets, live prey, or a combination, should be studied in terms of their impact on growth, flesh quality, especially proximate composition, fatty acid and amino acid profiles, molting, and overall health. Since blue crabs undergo multiple molts during their life cycle, understanding how diet affects the molting process is particularly important for soft-shell crab production, which requires precise timing of feed inputs to ensure crabs molt efficiently and consistently.

The goal of this study is to evaluate the effects of different diets on the growth performance, overall nutritional composition, and amino acid and fatty acid profiles of the blue crab (*Callinectes sapidus*) in a recirculating aquaculture system. By examining how various diets influence these factors, the study aims to provide valuable insights into optimizing feeding strategies for soft-shell blue crab production, with particular emphasis on improving both the quality and efficiency of growth. The findings will contribute to the development of more effective and sustainable aquaculture practices and high-performance diets for this commercially important species. To address this aim, we posed the following research question: How do different diets—commercial formulated feed (CF), minced trash fish (MTF), and a mixture of both (MIX)—affect the growth performance and molting frequency of blue crab under RAS conditions? We hypothesized that the MIX diet would yield superior growth and molting outcomes compared to either CF or MTF alone, due to a more balanced nutritional profile.

## 2. Materials and Methods

### 2.1. Ethics

Optimal rearing conditions were carefully maintained, and all necessary measures were taken to reduce animal suffering throughout the study. The feeding trial was conducted in compliance with the European Directive 2010/63/EU, which governs the protection of animals used in scientific research. As *Callinectes sapidus* is an invertebrate, no special permit was required under the prevailing legal framework.

### 2.2. Experimental Animals

A total of 300 *Callinectes sapidus* individuals, which are invasive in Türkiye, were collected from Lake Gala National Park (Turkish: Gala Gölü Milli Parkı) in Edirne Province, Northern Aegean Sea, on 7 November 2023. The crabs were harvested from Lake Pamuklu and Lake Küçük Gala, located within the boundaries of İpsala and Enez districts. At the time of crab sampling in Gala Lake, the water exhibited a pH of 7.51, a dissolved oxygen (DO) concentration of 6.77 mg/L, a salinity of 25 ppt, and a temperature of 12.5 °C.

Following collection, the crabs were transported to the laboratory under aerated conditions in insulated containers to minimize stress. Upon arrival, they were placed in a recirculating aquaculture system (RAS) for an acclimation period of 10 days. The system was maintained under sterile and controlled conditions, including temperature (23–24 °C), salinity (24–25 ppt), dissolved oxygen (>9 mg/L), and photoperiod (12:12 light/dark cycle).

At the end of the acclimation period, all individuals were weighed using a precision electronic balance (accuracy: 0.01 g), and carapace length and width were measured using digital calipers (accuracy: 0.1 mm). Following these measurements, 96 crabs with full extremities were randomly assigned to experimental groups (a total of 12 tanks, each containing 8 individuals). Five individuals were sacrificed for the initial proximate, amino acids, and fatty acid composition analysis.

### 2.3. Experimental Design

A 60-day experiment was conducted using a controlled recirculating aquaculture system (RAS) consisting of 12 circular tanks. Each group of four tanks was assigned to one experimental treatment, with each group connected to an independent RAS loop. Each tank had a volume of 650 L, with a radius of 95 cm and a depth of 1 m.

The filtration system included the following:

- A mechanical filter (sand filter; AFM, DrydenAqua, Edinburgh, UK) to remove suspended solids;
- A biological filter (moving bed biofilm reactor—MBBR (ADEC LSS, 2023, Istanbul, Türkiye); 0.96 m diameter × 1.5 m height, water depth 1.3 m) to support nitrifying bacterial communities for the conversion of ammonia and nitrite; and
- A UV sterilizer (UltraAqua, 40 mJ/cm^2^, 4.5 m^3^/h flow rate, 2023, Aalborg Øst, Denmark) for disinfection.

The system was designed to provide a complete water turnover approximately eight times per day for each tank. Backwashing of the sand filter was performed every four days, and only water lost during backwashing and surface splashing was replaced with clean water; no routine water exchange was conducted.

Before initiating the adaptation and experimental phases, the moving bed biofilm reactor (MBBR) units were biologically activated using a commercial bacterial culture (Seachem Stability^®^, 200 mL/system, 2023, Madison, GA, USA), which introduced nitrifying bacteria to establish the microbial community necessary for efficient ammonia and nitrite conversion. This pre-conditioning step was carried out over a 10-day period to ensure the biological filtration was fully functional.

Water quality parameters were monitored daily using a multi-parameter probe (HANNA HI9829, 2023, Woonsocket, RI, USA). The parameters measured included temperature (°C), pH, dissolved oxygen (DO, mg/L), salinity (ppt), and nitrate (NO_3_^−^, mg/L). Measurements were taken from each tank in the early morning to ensure stable and consistent water quality conditions throughout the experiment.

The experimental design included three dietary treatment groups, each with four replicates (*n* = 4). Each treatment group was assigned to a separate RAS unit, with each unit connected to four tanks to ensure independent replication. Within each tank, eight crabs were housed in individual perforated containers (15 cm width, 15 cm height, and 35 cm length) to prevent aggressive interactions. This setup resulted in a total of 32 crabs per dietary treatment group. Diets were prepared and provided to the experimental groups daily (3% g/bw) to evaluate their effects on growth performance and physiological responses. Uneaten feed was removed from the containers if refused.

### 2.4. Growth Parameters

Weight Gain (WG, g): The increase in body weight over the experimental period, calculated as the difference between the final and initial individual weights of the crabs, is expressed using the formulas below. Growth performance was assessed using weight gain (WG), carapace length gain (CLG), and carapace width gain (CWG) as described in previous studies [37,38].

WG = W_f_ − W_i_

where W_f_ is the final weight (g) and W_i_ is the initial weight (g) of the individual.

Carapace Length Gain (CLG, mm): The increase in carapace length over the course of the experiment was calculated as follows:

CLG = CL_f_ − CL_i_

where CL_f_ is the final carapace width (mm) and CL_i_ is the initial carapace width (mm).

Carapace Width Gain (CWG, mm): The increase in carapace width during the experiment was calculated as follows:

CWG = CW_f_ − CW_i_

where CW_f_ is the final carapace width (mm) and CW_i_ is the initial carapace width (mm).

All zootechnical measurements, including initial weight, final weight, and individual feed intake, were conducted individually for each crab. While crabs were housed in 4 replicate tanks per diet, each tank contained 8 individuals that were physically separated from each other to allow for individual monitoring and prevent cannibalism. Statistical analysis was performed on the individual crab data, recognizing that 8 individuals were housed in each of the 4 replicate tanks per diet.

Mortality was recorded daily. For the calculation of performance indices such as specific growth rate (SGR) and feed conversion ratio (FCR), only surviving individuals were included in the final biomass measurements. Feed intake was accounted for by summing the daily consumption of each live individual.

### 2.5. Experimental Diets

Three diet groups were used during the 60-day feeding trial: (1) Commercial Feed (CF), pelleted sea bass feed (No. 4, Çamlı); (2) Minced Trash Fish (MTF), composed of locally sourced low-value marine species; and (3) Mixed Diet (MIX), prepared by homogenizing ground commercial pellets and minced trash fish at a 1:1 ratio by wet weight.

For the MTF and MIX groups, low-value marine fish were purchased fresh from the Istanbul Fish Market. Two commonly available species—whiting (Merlangius merlangius euxinus, mezgit) and horse mackerel (Trachurus trachurus, istavrit)—were used in equal proportions (50:50 by weight). These fish were thoroughly washed, minced using a food-grade grinder, portioned, and stored in a freezer at −20 °C. Each portion was thawed and weighed before use.

All crabs were individually weighed at the start of the trial and every 15 days thereafter. The daily feed ration was calculated as 3% of individual body weight based on the most recent measurements. Feed was offered twice daily (09:00 and 15:00) by hand. Uneaten feed was removed after visual confirmation that the crab had stopped feeding. This approach ensured consistent and precise feed allocation across all individuals and prevented water quality degradation.

#### 2.5.1. Proximate Composition

The proximate composition of the experimental diets and crab muscle tissues was analyzed following standard methods of the Association of Official Analytical Chemists (AOAC). For each diet type, a single composite sample was prepared and analyzed in triplicate for moisture, crude protein, crude lipid, and ash contents (Table 1).

At the end of the feeding trial, 10 crabs were randomly selected from each dietary group. Muscle tissues from these individuals were pooled into a composite sample per group, which was then analyzed in triplicate for the same proximate parameters.

The samples were dried in an oven (DAIHAN Scientific, Wonju, Korea) at 105 °C until a constant weight was reached to determine the moisture content [39], while the ash content was determined by burning in a muffle furnace (Protherm FURNACES, Ankara, Türkiye) at 550 °C [40]. The crude protein content of the samples was determined using a semi-automatic Kjeldahl distillation system (Gerhardt Vapodest, 45S, Gerhardt, Germany), according to the Kjeldahl method [41]. The crude lipid content of the samples was determined by ether extraction using the Soxhlet system (Velp Scientifica Ser, 148, Milano, Italy) [42].

#### 2.5.2. Fatty Acid Analysis

Lipid extraction from both diets and crab meat was performed following the method described by Folch et al. (1957) [43], using a 2:1 chloroform/methanol solution for homogenization. The extracted lipids were then subjected to fatty acid methyl ester (FAME) synthesis using a modified version of the Ichihara et al. (1996) method [44]. For transmethylation, 10 mg of extracted lipid was dissolved in 2 mL of hexane, followed by the addition of 4 mL of 2M methanolic KOH. The mixture was vortexed for 2 min at room temperature and then centrifuged at 4000 rpm for 10 min. The resulting supernatant was used for gas chromatography (GC) analysis to determine fatty acid composition.

Fatty acid profiling was conducted using a Shimadzu GC-FID 2010 (Kyoto, Japan) system equipped with a 60 × 0.25 mm capillary column (Agilent DB-23, Santa Clara, CA, USA). Helium was used as the carrier gas, with the flame ionization detector (FID) set at 250 °C. The GC method parameters included a split ratio of 1:0, an injector temperature of 250 °C, and an oven temperature program starting at 120 °C for 2 min, increasing to 240 °C at a rate of 5 °C per minute over 7 min. Individual fatty acid methyl esters were identified by comparing retention times to commercial standards (Sigma-Aldrich, Steinheim, Germany). All analyses were conducted in triplicate to ensure accuracy and reproducibility (Table 2).

#### 2.5.3. Amino Acid Analysis

Amino acid composition was determined in both diets and crab meat (Table 3) following the procedures outlined by AOAC (2006) [45]. The analysis was performed using a high-sensitivity, high-speed triple quadrupole mass spectrometer (LCMS/8050, SHIMADZU system, Tokyo, Japan).

To extract amino acids, 1 g of the sample was mixed with 10 mL of petroleum ether and vortexed for 2 min to remove lipids. The mixture was then centrifuged for 3 min, and the upper petroleum ether layer was discarded. Subsequently, 25 mL of 6N HCl was added, and the mixture was transferred into a 250 mL Schott bottle. Hydrolysis was conducted in two steps: first, the sample was heated at 110 °C in a preheated oven for 1 h with the bottle uncovered, then the bottle was sealed and left to hydrolyze for an additional 23 h.

After hydrolysis, the sample was filtered through a 0.45 μm filter, transferred into a 15 mL Falcon tube, and diluted 1500 times before analysis. Amino acid quantification was carried out using LC-MS/MS, with each sample analyzed in triplicate to ensure accuracy and reproducibility.

### 2.6. Statistical Analysis

Proximate, amino acid, and fatty acid analyses were performed in triplicate, and the results were presented as mean  ±  standard deviation. Statistical analyses were conducted using IBM SPSS Statistics software (Version 30). The Shapiro–Wilk test was used to assess the normality of the data, and percentage data were arcsine-transformed before analysis. If the assumption of homogeneity of variance was met, a one-way ANOVA was applied. Tukey’s multiple range test was used for post hoc comparisons to determine differences between groups at a significance level of *p*  <  0.05.

Results of growth parameters and survival are presented as mean ± SE for each group. The homogeneity of variance was assessed using Levene’s test. Parametric one-way ANOVA was applied to compare initial weight, final weight, carapace width (CW), and carapace length (CL) values, followed by Tukey’s Honest Significant Difference (HSD) test for multiple comparisons among groups. A paired *t*-test was used to analyze the changes in Carapace Width, Carapace Length, and Weight before and after ecdysis events. For survival rate, the non-parametric Kruskal–Wallis test was used, followed by a multiple comparison test of mean ranks for all groups [46]. Statistical significance was set at *p* < 0.05. All analyses were performed using Statistica 8 software.

## 3. Results

### 3.1. Biometric Measurements and Survival Rates

#### 3.1.1. Growth Performance

The initial and final biometric measurements for the CF, MTF, and MIX groups are presented in Table 4. Statistically significant differences were observed among the experimental groups in terms of weight gain, carapace width (CW), and carapace length (CL). The final weight was significantly higher in the CF and MIX groups compared to the MTF group (*p* < 0.05). Similarly, the final carapace width values in the CF and MIX groups were significantly higher than the initial values of the same group (*p* < 0.05). However, no statistical difference was found in the MTF group (*p* > 0.05). In terms of carapace length, the CF and MIX groups again showed significantly greater final values than the MTF group (*p* < 0.05).

#### 3.1.2. Survival Rates

**Figure 1:**
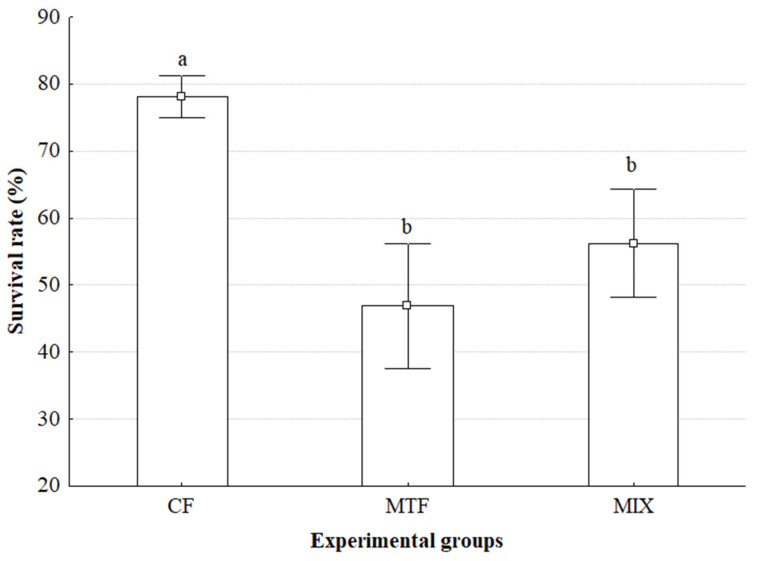
Survival rates (%) across experimental groups. In the experimental groups, CF: only commercial pellets; MTF: minced trash fish; MIX: pellets and minced trash fish mixed at a ratio of 50:50 were used as feed. ^a,b^ Means within the same column without common superscripts are significantly different (*p* < 0.05). Data are expressed as mean ± SE (*n* = 4).

The survival rate varied significantly across the experimental groups (Figure 1). CF exhibited the highest mean survival rate, which was significantly higher than both MTF and MIX, as indicated by the distinct letters above the bars (*p* < 0.05). While the survival rates of MTF and MIX were numerically different, they were not found to be statistically different from each other (*p* > 0.05). Statistical analysis using ANOVA revealed a significant effect of the experimental groups on survival rate (*p* < 0.05). Consistent results were obtained from the non-parametric Kruskal–Wallis H test (*p* < 0.05), further supporting the conclusion that the experimental groups had a significant impact on survival.

#### 3.1.3. Ecdysis Events

**Figure 2:**
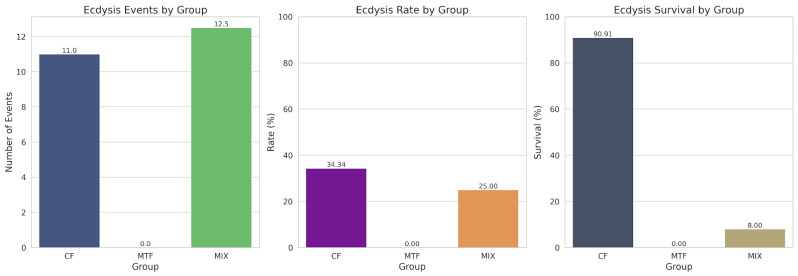
Total ecdysis events, rates, and survival across experimental groups.

In our study examining ecdysis patterns across three experimental groups, we observed substantial differences in molting frequency and survival outcomes (Figure 2). CF demonstrated superior performance across all measured parameters compared to MTF and MIX (*p* < 0.05). Specifically, CF exhibited 11 ecdysis events with a rate of 34.38%, representing a 37.50% increase over MIX (eight events, 25.00% rate). Most notably, the ecdysis survival rate in CF reached 90.91%, which was dramatically higher (627.28% increase) than MIX’s survival rate of 12.50% (*p* < 0.05). MTF showed no ecdysis activity throughout the experimental period. Descriptive statistics revealed considerable variability across groups (ecdysis count: mean = 6.33, SD = 5.69; ecdysis rate: mean = 19.79%, SD = 17.77%; survival rate: mean = 34.47%, SD = 49.28%).

*T*-tests show that the changes in carapace width, carapace length, and weight after ecdysis are statistically significant (*p* < 0.05) in CF. The very small *p*-values, particularly for CL and W, support that the crabs grew significantly during ecdysis.

**Figure 3:**
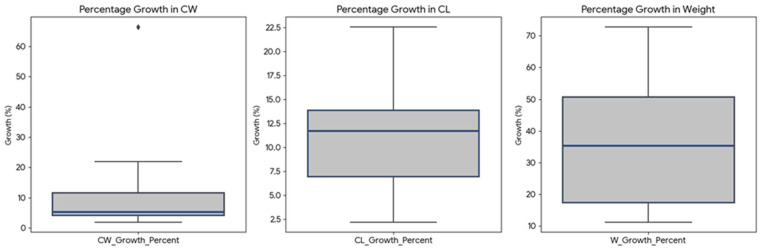
Percentage growth for carapace width, length, and weight before and after ecdysis in CF. The diamond shape highlights an outlier. This point represents one crab that experienced exceptionally high growth in its carapace width, a rate significantly greater than the rest of the crabs in the study.

All crabs increased in size (CW, CL) and weight (W) after ecdysis in CF. On average, carapace width increased by about 9.7 mm (12.7%), carapace length by 5.75 mm (11%), and weight by 31.2 g (36.2%). Percentage growth was most substantial in weight, followed by carapace width and carapace length (Figure 3), on average. There is considerable individual variability in growth rates for all parameters, especially for carapace width and weight. Some individuals experienced much larger percentage increases than others.

### 3.2. Chemical Analysis

#### 3.2.1. Proximate Composition

The proximate composition of crab meat across different experimental groups is summarized as follows: The experimental groups showed no significant differences compared to the initial values (*p* > 0.05). MIX and MTF had significantly higher lipid content than the initial values (*p* < 0.05), but CF was not significantly different from either. Moisture content was significantly lower in MTF compared to the initial value (*p* < 0.05), while MIX and CF retained significantly higher moisture levels than MTF. Ash content decreased in MTF, while MIX and CF showed intermediate values with no significant differences from either the initial or MTF values (Table 5).

#### 3.2.2. Fatty Acid Composition of Blue Crab Meats

The fatty acid composition of the experimental groups showed significant differences across various parameters (*p* < 0.05) (Table 6). Among saturated fatty acids (SFAs), palmitic acid (16:0) was the most abundant, with significantly higher levels in MTF and MIX compared to the initial and CF (*p* < 0.05). Stearic acid (18:0) also showed an increasing trend across the groups, with the highest level in CF. The total SFA content was significantly higher in MTF and MIX than in the initial and CF (*p* < 0.05). For monounsaturated fatty acids (MUFAs), oleic acid (18:1n-9) was the most dominant, with the highest concentration in the initial and the lowest in MTF. The total MUFA content was significantly reduced in the experimental groups compared to the initial, with the lowest value in MTF (*p* < 0.05). Polyunsaturated fatty acids (PUFAs) showed significant variations. Linoleic acid (LA, 18:2n-6) and arachidonic acid (ARA, 20:4n-6) increased significantly in the experimental groups, with the highest total n-6 PUFA content in CF (*p* < 0.05). Conversely, eicosapentaenoic acid (EPA, 20:5n-3) and docosahexaenoic acid (DHA, 22:6n-3) were significantly reduced in all experimental groups compared to the initial (*p* < 0.05), leading to a significant decrease in the total n-3 PUFA content. The highly unsaturated fatty acids (HUFAs) also followed a similar decreasing trend. The n-3/n-6 ratio was highest in the initial and significantly decreased across the experimental groups, with the lowest value in CF (*p* < 0.05). The DHA/EPA ratio, however, was significantly higher in CF, MTF, and MIX than in the initial, with the highest value in MTF (*p* < 0.05). The experimental treatments led to an increase in total SFA and n-6 PUFA content while reducing MUFAs and n-3 PUFA levels, particularly EPA and DHA.

#### 3.2.3. Amino Acid Composition of Blue Crab Meat

The amino acid composition of the experimental groups exhibited significant variations (*p* < 0.05) (Table 7). Among essential amino acids (EAAs), arginine, isoleucine, leucine, threonine, and valine levels significantly decreased in all experimental groups compared to the initial (*p* < 0.05). In contrast, histidine and lysine concentrations increased significantly in CF, MTF, and MIX. Methionine and phenylalanine levels varied among the groups, with CF and MTF treatments showing reductions compared to the initial value (*p* < 0.05). For non-essential amino acids (NEAAs), alanine, aspartic acid, and serine showed significant increases in the experimental groups, particularly with the highest alanine content observed in CF (*p* < 0.05). Cystine and hydroxyproline levels significantly decreased in all experimental groups (*p* < 0.05), while glutamic acid and glycine levels remained relatively stable (*p* > 0.05). Proline levels were significantly lower in all experimental groups compared to the initial (*p* < 0.05). The total EAA content significantly decreased in all experimental groups compared to the initial (*p* < 0.05). Similarly, total NEAA content and total amino acid content were significantly lower in the experimental groups (*p* < 0.05). The EAAs/NEAAs ratio also decreased, with the lowest value recorded in CF (*p* < 0.05).

## 4. Discussion

### 4.1. Growth Parameters

This study demonstrated that dietary regimen significantly impacted the growth performance of *Callinectes sapidus*, with crabs fed commercial feed (CF) and a 50:50 mixture (MIX) showing superior growth metrics (final weight, carapace width, and length) compared to those on a minced trash fish (MTF) diet.

The experiment’s design ensured all crabs were individually housed in separate containers, eliminating social interactions such as competition or cannibalism. This setup minimized external behavioral influences on feeding, stress, and molting, providing a controlled assessment of dietary effects. However, it may also have reduced behavioral stimuli known to influence molting rhythms and activity levels in decapods.

A central biological factor influencing these outcomes is ecdysis, or molting, which is essential for growth in crustaceans. Crustaceans grow discontinuously, with increases in somatic size occurring exclusively after successful molts [47]. If molting is limited in frequency or completeness, no observable increase in parameters such as body weight, CW, or CL can take place, regardless of dietary composition.

In this study, the lack of significant growth observed in the MTF group can be directly attributed to a complete absence of ecdysis throughout the experimental period. This strongly suggests that the minced trash fish diet was insufficient to support the physiological conditions required for successful molting. A key contributing factor to this deficiency was likely the substantially lower dry matter content of the MTF diet (30.58%) compared to the commercial feed (CF) (91.45%). Despite equal wet weight feeding, this significant difference in dry matter almost certainly resulted in a markedly lower actual nutrient intake in the MTF group. This overall reduced nutrient availability, compounded by potential degradation during storage and handling of raw fish, likely rendered the MTF diet inadequate to meet the high energetic and specific micronutrient demands (e.g., cholesterol, calcium–phosphate balance) essential for initiating and completing the complex molting cycle [47].

Our findings are consistent with earlier work suggesting that the influence of diet on growth is highly dependent on molting frequency. For example, Catacutan (2002) [48] demonstrated that juvenile *Scylla serrata* exhibited optimal growth when fed diets containing 32–40% protein with varying lipid levels, yet these effects were only apparent under conditions that allowed for consistent molting. Similarly, Unnikrishnan and Paulraj (2010) [49] found that a 47% dietary protein level was optimal for growth in *S. serrata* juveniles, further highlighting the importance of nutritional adequacy during molting phases.

Our results also parallel the general observation that different dietary compositions elicit varying growth responses in *Callinectes sapidus*. For instance, Gencer and Vitorino (2023) [20] observed significant differences in the growth performance of first crab stage *C. sapidus* juveniles fed natural diets (shrimp, squid, tilapia). While their specific optimal diet (shrimp) may differ from our findings due to variations in diet formulation, crab life stage, or experimental conditions, their work similarly supports that protein source and quality can significantly influence crab growth when molting occurs regularly.

The varying growth, survival, and molting performance across our dietary groups underscore the profound influence of feed composition and quality on *C. sapidus* in RASs. While the MIX diet supported slightly higher growth metrics, the Commercial Feed (CF) diet yielded optimal survival and ecdysis frequency, indicating a more balanced nutritional profile for overall performance. This aligns with recent studies by Gencer (2025) [21], who observed superior growth and survival in *C. sapidus* with optimized marine-based diets, and Conti et al. (2024) [35], who highlighted the sensitivity of *C. sapidus* growth to even subtle differences in dietary fatty acids (e.g., marine vs. terrestrial sources). Our findings, alongside others such as of Belgrad and Griffen (2016) [25], who noted higher mortality in crabs fed seaweed compared to animal tissue, challenge the assumption that all ‘natural’ diet components are equally beneficial, emphasizing the need for nutritionally complete and balanced formulations.

The observed differences in performance among the dietary groups can be attributed to variations in feed digestibility and the subsequent metabolic utilization of nutrients. An optimal balance of macronutrients (protein, lipid, carbohydrate) and micronutrients (vitamins, minerals, fatty acids) is critical, as it directly governs the organism’s ability to extract and allocate energy for essential physiological processes, particularly those supporting growth through successful molting.

#### Digestibility, Nutrient Availability, Metabolic Variation, and Energy Allocation

The observed dietary effects on growth, survival, and molting are intricately linked to feed digestibility and subsequent metabolic energy allocation. While *C. sapidus*, as opportunistic omnivores, exhibit adaptable digestive enzyme profiles [50], the efficiency of nutrient assimilation is highly sensitive to feed composition. The physical properties of a diet (e.g., palatability, particle size) further influence ingestion rates, setting the stage for nutrient uptake.

This explains why the MIX diet, potentially offering more readily digestible components or a balanced amino acid and fatty acid profile, may have supported slightly higher growth metrics. Conversely, the Commercial Feed (CF), perhaps optimized for the digestibility of elements crucial for immunity or cuticle formation, could have contributed to superior survival and molting frequency. As Belgrad and Griffen (2016) [25] demonstrated in crabs, animal-based diets lead to higher consumption and lower mortality compared to plant-based alternatives, suggesting enhanced nutrient uptake and utilization critical for overall fitness.

Beyond simple digestibility, different diets impose varying metabolic loads, influencing how energy is partitioned among vital functions such as growth, maintenance, molting, and immunity. Lipid quality, especially fatty acid profiles, is paramount in crustacean metabolism. Conti et al. (2024) [35] showed that diets with imbalanced fatty acid profiles (e.g., ‘terrestrial’ diets low in n-3 PUFAs) significantly reduced *C. sapidus* body weight, illustrating how suboptimal lipid sources impair growth even with sufficient caloric intake. Their work also suggested potential trade-offs, such as prioritizing reproductive investment overgrowth under nutritional stress, which could explain differing outcomes in survival versus growth across diets. This could explain why one diet might favor survival (perhaps by boosting immune function) while another favors growth, depending on how metabolic energy is allocated. Molting itself is a highly energy-demanding process, and a diet providing readily available energy and specific precursors (e.g., for chitin synthesis) would efficiently support more frequent and successful molting cycles. This efficiency in nutrient provision and bioavailability by the CF diet likely contributed to the increased ecdysis frequency observed.

A crucial factor influencing comparative performance was likely the handling and preservation of the trash fish used in the MTF and MIX diets. Although initially fresh, prolonged freezing at −20 °C and repeated thawing can lead to significant degradation of nutritional quality, especially through lipid oxidation and loss of heat-sensitive nutrients. This diminished feeding value likely undercuts the potential benefits of these ‘natural’ protein sources compared to the nutritionally stable and well-balanced commercial pellets.

Indeed, the superior performance of the CF group highlights the advantages of modern commercial pellet formulations. These feeds offer enhanced digestibility, precise micronutrient balance, and superior hygiene, which are particularly beneficial in controlled RAS environments. Our findings thus emphasize that while natural or mixed diets can support some growth, consistent quality, bioavailability, and biosecurity of commercial feeds offer distinct advantages in key aquaculture parameters such as survival and consistent molting frequency.

In conclusion, our results strongly indicate that experimental diets significantly impact the growth performance of *C. sapidus* in RASs. These outcomes consistently underscore molting as the indispensable physiological gateway to growth in crabs, emphasizing that any successful nutritional strategy must actively support consistent ecdysis. Future research should consider extending rearing periods to observe multiple molt cycles, incorporate detailed molt-stage monitoring, and explore environmental optimizations to further delineate dietary effects. Additionally, investigating alternative protein sources or functional feed additives, as shown in other decapod species [51], holds promise for developing more effective and sustainable diets for blue crab aquaculture.

### 4.2. Feeding and Water Quality

Our study demonstrates that effective water quality management in a recirculating aquaculture system (RAS) is crucial for successful *Callinectes sapidus* culture, especially when utilizing varying dietary protein levels. Consistent feeding, coupled with robust biofiltration via an activated moving bed biofilm reactor (MBBR) and low stocking density, successfully maintained water parameters within optimal ranges throughout the 60-day trial. This finding aligns with studies on other crab species, such as *Scylla* spp. [52,53,54], which highlight how stable RAS environments mitigate ammonia accumulation, a common concern with high-protein diets in intensive aquaculture.

The superior growth performance and health observed in our RAS-cultured *C. sapidus* are consistent with findings by Kohinoor et al. (2019) [55] on juvenile blue swimming crabs (*Portunus pelagicus*). Their work demonstrated that RASs provide more stable and optimal water quality parameters compared to conventional systems, directly contributing to improved crustacean health and growth. Similarly, Nurcahyono et al. (2022) [56] observed that well-maintained biological filters in RASs could optimize water quality for female reproductive success in mud crabs. This further corroborates our finding that a well-adapted MBBR effectively sustained the optimal water quality conditions necessary for the successful culture of *C. sapidus*.

### 4.3. Survival Rates

Our study revealed significant variations in survival rates among the experimental diets, with the Commercial Feed (CF) group demonstrating substantially higher survival (≈78%) compared to the Minced Trash Fish (MTF) (≈47%) and Mixture (MIX) (≈56%) diets. This stark difference indicates that beyond influencing growth metrics, dietary composition profoundly affects the physiological resilience and overall health status of *Callinectes sapidus*, directly impacting their survivability in a controlled environment.

The literature on *C. sapidus* culture in Recirculating Aquaculture Systems (RASs) is limited, particularly for later juvenile and adult stages, due to the inherent challenges of rearing older crabs under controlled conditions [20]. Most nutritional studies have focused on early crab stages, which are more manageable in terms of space and feeding uniformity. Gencer and Vitorino (2023) [20], for instance, reported higher survival in first-crab stage *C. sapidus* fed shrimp-based diets compared to those on squid or tilapia, suggesting that protein source and quality are critical for crab vitality and robustness in culture.

While our findings align with the importance of diet quality, they present an interesting contrast to some earlier work. Suprapto (2001) [57] found that *Scylla serrata* juveniles exhibited significantly better survival on fresh fish diets compared to other regimes, emphasizing the positive impact of natural feed and digestibility on survival under intensive rearing. Furthermore, environmental factors, such as salinity, can interact with nutrition to influence survival in *S. serrata* crablings [58]. Our study, by assessing survival in later juvenile stages under individualized, socially isolated conditions, provides unique insights into the direct effects of diet, free from confounding social stressors such as aggression or cannibalism common in group housing.

The superior survival observed in the CF group strongly suggests that its nutritional profile better supported stress resistance and immune function. This highlights a critical need for further research into diet optimization, not only for growth but also for enhancing survival in cultured *C. sapidus* beyond the larval stage. Understanding these specific dietary components will be crucial for developing more robust and successful aquaculture practices.

### 4.4. Molting Frequency and Survival Outcomes

The pronounced differences in both molting frequency and survival rates among our experimental groups critically underscore the central role of ecdysis in the growth and viability of *C. sapidus*. The Commercial Feed (CF) diet yielded the highest molting frequency concurrently with the highest survival rate, strongly suggesting that its specific dietary formulation effectively supported the physiological processes essential for successful molting and overall resilience.

Molting in crustaceans is a complex, hormonally regulated process, primarily governed by ecdysteroids, which initiate ecdysis. The synthesis and release of these hormones are significantly influenced by nutritional status. Adequate nutrient intake can modulate the levels of molt-inhibiting hormone (MIH), thereby facilitating the progression of the molting cycle [47]. In our study, the enhanced molting frequency observed in the CF group likely reflects a dietary composition that effectively modulated these hormonal pathways, promoting more consistent and successful ecdysis.

Similar interplays between environmental factors and molting success have been reported in other crustacean species. For instance, Fatihah et al. (2017) [59] observed increased molting frequency and survival in *Scylla tranquebarica* with specific shelter conditions, and Zhang et al. (2022) [60] demonstrated the influence of dietary composition, salinity, and light on molting and survival in *Scylla paramamosain*.

The complete absence of molting activity in the MTF group throughout the experimental period further reinforces the critical importance of an appropriate and nutritionally sufficient dietary formulation in supporting the molting process. Without successful ecdysis, crustaceans cannot grow, and their long-term survival is severely compromised [47]. This finding underscores that continuous molting is a fundamental prerequisite for *C. sapidus* development and that any disruption to this process has detrimental effects on both growth and survival.

The substantial differences observed, particularly in survival outcomes, strongly suggest a biologically significant effect of our dietary interventions. While the limited sample size precluded the application of traditional inferential statistics, the sheer magnitude of the observed differences, especially in the CF group’s superior survival, warrants further investigation with larger sample sizes to confirm these trends and to elucidate the underlying mechanisms by which specific dietary components influence molting and survival in *C. sapidus*.

### 4.5. Proximate Composition, Amino Acid, and Fatty Acid Composition of Crab Meat

Dietary interventions significantly influenced the biochemical composition of *Callinectes sapidus* meat. While protein content remained largely stable across all experimental groups, falling within established blue crab ranges (15–19%) [61], other components exhibited distinct shifts. Lipid content increased significantly in the MTF and MIX groups compared to CF and initial values, aligning with expected variability influenced by diet [62,63,64,65,66]. Variations in moisture and ash content, particularly lower levels in MTF, further suggested differential water retention and mineral deposition influenced by diet. These proximate composition results underscore how feed formulation, even with subtle changes, can impact the nutritional quality and marketability of the harvested product.

Analysis of fatty acid profiles revealed significant alterations from the initial state, highlighting the sensitivity of blue crab lipid metabolism to dietary inputs. We observed a direct correlation between dietary saturated fatty acids (SFAs) and their accumulation in crab tissues, with higher ΣSFA in MTF and MIX, predominantly palmitic acid (16:0) [22,67,68]. Similarly, tissue monounsaturated fatty acid (MUFA) content, particularly oleic acid (18:1n-9), closely reflected dietary availability [69,70,71].

For polyunsaturated fatty acids (PUFAs), despite relatively stable dietary ΣPUFA across treatments, their composition within the crab tissues changed markedly. N-6 PUFAs, including linoleic (18:2n-6) and arachidonic acid (20:4n-6), significantly increased in experimental groups, mirroring their higher dietary supply in some feeds, particularly CF. Conversely, beneficial n-3 highly unsaturated fatty acids (HUFAs) such as EPA (20:5n-3) and DHA (22:6n-3) were significantly reduced in all experimental groups compared to the initial samples. This depletion directly correlated with their lower availability in the experimental diets compared to a presumed wild diet. Consequently, the n-3/n-6 ratio significantly decreased across all experimental groups, reaching its lowest in CF, directly reflecting the dietary ratios [72,73]. This ratio is a key indicator of nutritional quality for consumers, with a lower value generally being less favorable. The observed low n-3/n-6 ratio in CF could also partially result from the high metabolic demands of ecdysis, potentially involving preferential catabolism of n-3 fatty acids from stored reserves. The DHA/EPA ratio also increased in all experimental groups compared to initial values, suggesting differential retention, utilization, or conversion rates influenced by dietary proportions and the metabolic state related to molting [74,75,76].

Regarding amino acid composition, while the diets varied significantly in total amino acid (AA) content, tissues of experimental groups generally showed a net depletion of total and essential amino acids (EAAs) compared to the initial group, and an altered EAA/NEAA ratio. The MTF and MIX diets, with lower overall AA content and less balanced EAA/NEAA ratios, likely contributed to reduced tissue concentrations [28,77]. Key EAAs, such as arginine, isoleucine, leucine, threonine, and valine, decreased, suggesting that the experimental diets may not have fully met requirements, leading to depletion of tissue reserves. Conversely, some non-essential amino acids (NEAAs), such as alanine, aspartic acid, and serine, increased, potentially reflecting altered metabolic pathways or protein turnover [28]. The significant decrease in collagen components such as hydroxyproline and proline might indicate changes in connective tissue metabolism. The metabolic demands of ecdysis in the CF group (34% molting rate), a process requiring substantial protein synthesis for new cuticle and tissue formation [78,79,80], would significantly impact amino acid pools, potentially explaining the lower total amino acid content and altered proportions in CF despite its higher dietary amino acid content. In contrast, the lack of molting in MTF suggests its observed amino acid changes are more directly attributable to dietary input and general maintenance. These findings underscore the critical importance of adequate and balanced dietary amino acid supply, particularly EAAs, to support both maintenance and energetically demanding processes such as molting in *C. sapidus* [28,77].

## 5. Conclusions

This study demonstrates that the composition of diets significantly influences the biochemical and growth responses of *Callinectes sapidus* in a recirculating aquaculture system. Crabs fed with the commercial pellet (CF) and mixed (MIX) diets achieved significantly greater final weights and carapace sizes compared to those fed minced trash fish (MTF). This indicates superior somatic growth performance under the CF and MIX dietary regimes in this experimental setup. The observed growth differences are likely linked to improved nutrient assimilation and more frequent successful molting events.

Biochemical analyses revealed that dietary input was directly reflected in crab tissue profiles. CF and MIX groups exhibited lower n-3/n-6 ratios and altered DHA/EPA ratios compared to MTF. Essential amino acid concentrations were also generally reduced in all experimental groups compared to the initial state, particularly in groups undergoing active molting, suggesting increased metabolic demand. These results emphasize the importance of balancing essential nutrient content in formulated feeds to support optimal growth and physiological development during ecdysis.

From a practical perspective, our findings suggest that diets providing a higher concentration of available nutrients, such as the pellet-based (CF) or mixed (MIX) formulations, can lead to enhanced growth and improved nutritional quality of farmed blue crabs compared to a fresh, unsupplemented trash fish diet administered on an equal wet-weight basis. This is particularly evident given the lower dry matter content of the MTF diet, which likely contributed to reduced actual nutrient intake in that group. While this study did not compare iso-nutritionally balanced diets, the outcomes underscore that adequate nutrient density is paramount for supporting growth and molting in *C. sapidus*. Adoption of more nutritionally dense feeds in commercial aquaculture operations may improve feed management, potentially reduce environmental waste, and contribute to more sustainable and economically viable crab farming systems.

## Figures and Tables

**Table 1:** Proximate composition of diets used in experimental trial.

Proximate Composition	Groups ^1^		
	CF	MTF	MIX
Dry matter, %	92.17	31.79	61.98
Crude protein, %	46.65	16.09	31.70
Crude lipid, %	17.63	1278	15.15
Ash, %	8.94	2.36	5.69
Fiber, %	1.64	0.25	0.81
NFE ^a^, kJ/g	17.31	0.31	8.63

^1^ In the experimental groups, CF: only commercial pellets; MTF: minced trash fish; MIX: pellets and minced trash fish mixed at a ratio of 50:50 were used as feed. ^a^ NFE: Nitrogen-free extract.

**Table 2:** Fatty acid composition of diets used in experimental trial (% of total fatty acids) ^1^.

Fatty Acids	Feeds		
	CF	MTF	MIX
14:0	2.53	7.34	4.69
15:0	0.21	0.97	0.44
16:0	13.47	22.11	17.55
17:0	0.27	1.25	0.70
18:0	4.75	7.33	5.13
20:0	0.32	ND	0.13
24:0	1.01	ND	0.68
16:1n-7	3.63	7.49	5.87
18:1n-9	38.32	11.72	24.53
20:1n-9	2.90	5.70	4.17
22:1n-11	1.96	10.30	6.31
24:1n-9	0.40	ND	0.21
18:2n-6	15.12	2.15	8.34
20:2n-6	0.73	ND	0.68
18:3n-3	5.39	0.95	2.61
20:3n-3	0.15	ND	0.07
20:3n-6	0.40	ND	0.22
20:4n-6	0.28	0.90	0.46
20:5n-3	3.38	6.57	4.69
22:6n-3	4.05	15.23	9.95
ΣSFA ^a^	22.56	38.99	29.32
ΣMUFA ^b^	47.21	35.21	41.09
ΣPUFA ^c^	29.50	25.81	27.02
Σn-3 HUFA ^d^	7.58	21.80	14.71
Σn-3	12.97	22.75	17.32
Σn-6	16.53	3.05	9.70
n-3/n-6	0.78	7.46	1.79
DHA/EPA	1.20	2.32	2.12

^1^ In the experimental groups, CF: only commercial pellets; MTF: minced trash fish; MIX: pellets and minced trash fish mixed at a ratio of 50:50 were used as feed. ND: not detected. ^a^ SFAs (saturated fatty acids): 14:0, 15:0, 16:0, 17:0, 18:0, 20:0, and 24:0. ^b^ MUFAs (monounsaturated fatty acids): 14:1 to 24:1n-9. ^c^ PUFAs (polyunsaturated fatty acids): 18:2n-6 to 22:6n-3. ^d^ HUFAs (highly unsaturated fatty acids): 20:3n-3, 20:5n-3, and 22:6n-3.

**Table 3:** Amino acid composition of experimental diets used in the trial (g amino acid/100 g feed) ^1^.

Amino Acids (AAs)	Feeds		
	CF	MTF	MIX
Essential amino acids (EAAs)			
Arginine	1.52	0.76	1.22
Histidine	0.42	0.03	0.23
Isoleucine	6.11	2.47	4.49
Leucine	10.74	3.45	6.94
Lysine	2.45	0.91	1.65
Methionine	1.02	1.10	1.07
Phenylalanine	3.48	1.23	2.47
Threonine	0.03	0.11	0.10
Valine	4.18	1.08	2.83
Non-essential amino acids (NEAAs)			
Alanine	0.47	0.23	0.35
Aspartic Acid	0.12	0.03	0.06
Cystine	0.03	0.05	0.05
Glutamic Acid	7.57	0.50	3.8
Glycine	0.91	0.13	0.51
Hydroxyproline	2.98	1.61	2.46
Proline	3.27	2.34	3.11
Serine	0.06	0.06	0.07
Tyrosine	0.15	0.07	0.13
ΣΕAA	29.95	11.14	21.01
ΣΝΕAA	15.55	5.03	10.55
ΣAA	45.51	16.16	31.55
EAA/NEAA	1.93	1.49	1.79

^1^ In the experimental groups, CF: only commercial pellets; MTF: minced trash fish; MIX: pellets and minced trash fish mixed at a ratio of 50:50 were used as feed.

**Table 4:** Mean initial and final weight, CW, and CL data for blue crabs.

Parameters	Groups ^1^					
	CF		MTF		MIX	
	Initial	Final	Initial	Final	Initial	Final
Weight, g	102.32 ± 2.69 ^B^	115.64 ± 3.03 ^aA^	101.24 ± 2.73	102.81 ± 2.25 ^b^	100.15 ± 2.21 ^B^	113.53 ± 2.53 ^aA^
CW, mm	93.36 ± 1.08 ^B^	97.88 ± 1.02 ^A^	94.05 ± 1.48	95.24 ± 1.19	93.72 ± 0.57 ^B^	97.16 ± 0.43 ^A^
CL, mm	56.82 ± 1.21	58.84 ± 0.48 ^a^	55.26 ± 0.36	55.376 ± 0.67 ^b^	55.21 ± 0.56	57.30 ± 0.21 ^a^

^1^ In the experimental groups, CF: only commercial pellets; MTF: minced trash fish; MIX: pellets and minced trash fish mixed at a ratio of 50:50 were used as feed. ^a,b^ Letters on the same line indicate the difference between the final results of the groups in statistical comparisons. ^A,B^ Letters on the same line indicate the difference between the results within the group (Initial compared to Final) in statistical comparisons. CW: Carapace width, CL: Carapace length. Data are expressed as mean ± SD (*n* = 4).

**Table 5:** Initial and final proximate compositions of blue crabs.

Proximate Composition, %	Groups ^1^			
	Initial	CF	MTF	MIX
Protein	17.81 ± 0.17	16.29 ± 1.73	16.68 ± 1.81	16.61 ±1.76
Lipid	0.65 ± 0.02 ^b^	0.73 ± 0.05 ^ab^	0.78 ± 0.04 ^a^	0.79 ± 0.06 ^a^
Moisture	79.34 ± 0.08 ^a^	78.36 ± 0.39 ^a^	76.57 ± 0.46 ^b^	78.34 ± 0.09 ^a^
Ash	2.16 ± 0.04 ^a^	1.96 ± 0.08 ^ab^	1.87 ± 0.04 ^b^	1.96 ± 0.13 ^ab^

^1^ In the experimental groups, CF: only commercial pellets; MTF: minced trash fish; MIX: pellets and minced trash fish mixed at a ratio of 50:50 were used as feed. ^a,b^: Lowercase letters in each row indicate significant differences (*p* < 0.05) between groups. Data are expressed as mean ± SD.

**Table 6:** Initial and final fatty acid compositions of blue crabs (edible parts) fed the experimental diets (% of detected fatty acids).

	Groups ^1^			
Lipid and Fatty Acids	Initial	CF	MTF	MIX
Lipid (%)	0.65 ± 0.02 ^b^	0.73 ± 0.05 ^a^	0.78 ± 0.03 ^a^	0.79 ± 0.06 ^a^
14:0	1.74 ± 0.16 ^c^	2.97 ± 0.44 ^b^	3.24 ± 0.53 ^a^	3.29 ± 0.54 ^a^
15:0	0.68 ± 0.03 ^a^	0.61 ± 0.10 ^a^	0.38 ± 0.07 ^b^	0.39 ± 0.09 ^b^
16:0	18.65 ± 1.23 ^b^	19.56 ± 2.17 ^b^	24.96 ± 2.04 ^a^	25.54 ± 2.23 ^a^
17:0	0.66 ± 0.04 ^a^	0.65 ± 0.06 ^a^	0.43 ± 0.04 ^b^	0.43 ± 0.02 ^b^
18:0	4.80 ± 0.46 ^c^	7.89 ± 1.36 ^a^	6.74 ± 1.01 ^b^	6.85 ± 1.23 ^b^
20:0	ND	0.53 ± 0.06	0.48 ± 0.06	0.52 ± 0.05
24:0	1.92 ± 0.25 ^a^	0.35 ± 0.09 ^d^	1.55 ± 0.26 ^b^	1.16 ± 0.21 ^c^
15:1n-*cis*10	2.28 ± 0.57 ^a^	0.26 ± 0.03 ^c^	0.76 ± 0.19 ^b^	ND
16:1n-7	7.08 ± 0.67 ^a^	4.37 ± 0.93 ^b^	4.20 ± 0.82 ^c^	3.89 ± 0.77 ^c^
17:1n	1.42 ± 0.16 ^a^	0.22 ± 0.02 ^b^	0.21 ± 0.02 ^b^	0.22 ± 0.03 ^b^
18:1n-9	21.07 ± 1.47 ^a^	19.26 ± 1.91 ^b^	15.92 ± 1.71 ^d^	17.77 ± 1.62 ^c^
20:1n-9	0.95 ± 0.04 ^c^	2.75 ± 0.34 ^a^	1.84 ± 0.27 ^b^	1.91 ± 0.39 ^b^
22:1n-11	ND	0.29 ± 0.02 ^b^	0.54 ± 0.06 ^a^	0.50 ± 0.03 ^a^
24:1n-9	ND	0.56 ± 0.11 ^c^	3.04 ± 0.51 ^a^	2.35 ± 0.44 ^b^
18:2n-6	ND	4.90 ± 0.63 ^a^	2.88 ± 0.42 ^b^	3.16 ± 0.56 ^b^
20:2n-6	ND	0.43 ± 0.06 ^a^	0.13 ± 0.01 ^b^	0.12 ± 0.02 ^b^
18:3n-3	2.93 ± 0.36 ^a^	1.58 ± 0.19 ^b^	0.28 ± 0.02 ^c^	0.36 ± 0.04 ^c^
20:3n-3	ND	ND	ND	ND
20:3n-6	ND	0.11 ± 0.01	ND	ND
20:4n-6	4.59 ± 0.37 ^b^	6.21 ± 1.61 ^a^	6.07 ± 1.26 ^a^	6.06 ± 1.33 ^a^
20:5n-3	17.60 ± 1.03 ^a^	8.49 ± 1.29 ^b^	9.14 ± 1.75 ^b^	8.92 ± 1.66 ^b^
22:6n-3	7.23 ± 0.69 ^a^	5.96 ± 1.14 ^b^	7.26 ± 1.44 ^a^	6.20 ± 0.96 ^b^
ΣSFA ^a^	28.45 ± 1.48 ^c^	32.56 ± 2.08 ^b^	37.78 ± 2.01 ^a^	38.18 ± 2.10 ^a^
ΣMUFA ^b^	32.80 ± 1.71 ^a^	27.23 ± 1.83 ^b^	25.54 ± 1.89 ^c^	26.42 ± 1.82 ^bc^
ΣPUFA ^c^	32.32 ± 1.56 ^a^	27.68 ± 2.22 ^b^	25.76 ± 2.20 ^c^	24.82 ± 2.14 ^c^
Σn-3 HUFA ^d^	24.83 ± 1.31 ^a^	14.45 ± 1.56 ^c^	16.40 ± 1.79 ^b^	15.12 ± 1.62 ^bc^
Σn-3	27.76 ± 1.44 ^a^	16.03 ± 1.62 ^b^	16.68 ± 1.79 ^b^	15.48 ± 1.63 ^b^
Σn-6	4.59 ± 0.37 ^c^	11.65 ± 1.52 ^a^	9.08 ± 1.30 ^b^	9.34 ± 1.38 ^b^
n-3/n-6	6.04 ± 0.48 ^a^	1.38 ± 0.13 ^d^	1.84 ± 0.21 ^b^	1.65 ± 0.18 ^c^
DHA/EPA	0.41 ± 0.01 ^c^	0.79 ± 0.03 ^a^	0.70 ± 0.03 ^b^	0.70 ± 0.02 ^b^

^1^ In the experimental groups, CF: only commercial pellets; MTF: minced trash fish; MIX: pellets and minced trash fish mixed at a ratio of 50:50 were used as feed. ND: not detected. ^a–d^ Means ± SD followed by different letters within a row are significantly different (*p* < 0.05). Data are expressed as mean ± SD (*n* = 3). ^a^ SFAs (saturated fatty acids): 14:0, 15:0, 16:0, 17:0, 18:0, 20:0, and 24:0. ^b^ MUFAs (monounsaturated fatty acids): 14:1 to 24:1n-9. ^c^ PUFAs (polyunsaturated fatty acids): 18:2n-6 to 22:6n-3. ^d^ n-3 HUFAs (highly unsaturated fatty acids): 20:3n-3, 20:5n-3, and 22:6n-3.

**Table 7:** Initial and final amino acid compositions of blue crabs (edible parts) fed the experimental diets (g per 100 g of crude protein).

	Groups ^1^			
Protein and Amino Acids	Initial	MTF	MIX	CF
Protein (%)	17.81 ± 0.17 ^a^	16.68 ± 1.81 ^a^	16.61 ± 1.76 ^a^	16.29 ± 1.73 ^a^
Essential Amino Acids (EAAs)				
Arginine	1.13 ± 0.09 ^a^	0.61 ± 0.04 ^b^	0.56 ± 0.05 ^b^	0.52 ± 0.04 ^b^
Histidine	0.42 ± 0.04 ^d^	0.67 ± 0.06 ^a^	0.60 ± 0.06 ^b^	0.50 ± 0.05 ^c^
Isoleucine	0.99 ± 0.08 ^a^	0.88 ± 0.11 ^b^	0.81 ± 0.10 ^b^	0.83 ± 0.10 ^b^
Leucine	1.67 ± 0.12 ^a^	1.48 ± 0.20 ^b^	1.43 ± 0.19 ^b^	1.44 ± 0.19 ^b^
Lysine	1.35 ± 0.10 ^b^	1.77 ± 0.19 ^a^	1.72 ± 0.19 ^a^	1.76 ± 0.19 ^a^
Methionine	0.53 ± 0.05 ^a^	0.41 ± 0.03 ^b^	0.53 ± 0.05 ^a^	0.47 ± 0.03 ^b^
Phenylalanine	0.81 ± 0.07 ^a^	0.61 ± 0.07 ^c^	0.72 ± 0.07 ^b^	0.58 ± 0.04 ^c^
Threonine	1.03 ± 0.08 ^a^	0.58 ± 0.04 ^b^	0.56 ± 0.03 ^b^	0.57 ± 0.04 ^b^
Valine	0.95 ± 0.08 ^a^	0.55 ± 0.06 ^b^	0.51 ± 0.05 ^b^	0.49 ± 0.05 ^b^
Non-essential Amino Acids (NEAAs)				
Alanine	0.85 ± 0.07 ^c^	1.19 ± 0.11 ^b^	1.21 ± 0.12 ^b^	1.31 ± 0.12 ^a^
Aspartic acid	1.71 ± 0.13 ^b^	1.71 ± 0.04 ^b^	1.66 ± 0.03 ^c^	1.80 ± 0.03 ^a^
Cystine	0.17 ± 0.01 ^a^	0.07 ± 0.01 ^b^	0.11 ± 0.01 ^b^	0.08 ± 0.01 ^b^
Glutamic acid	2.63 ± 0.18	2.42 ± 0.05	2.35 ± 0.04	2.46 ± 0.05
Glycine	1.25 ± 0.10 ^a^	1.24 ± 0.07 ^a^	1.17 ± 0.07 ^a^	1.08 ± 0.05 ^b^
Hydroxyproline	0.65 ± 0.06 ^a^	0.42 ± 0.04 ^b^	0.44 ± 0.05 ^b^	0.33 ± 0.04 ^c^
Proline	0.84 ± 0.07 ^a^	0.45 ± 0.05 ^b^	0.48 ± 0.05 ^b^	0.47 ± 0.05 ^b^
Serine	0.84 ± 0.07 ^b^	0.94 ± 0.06 ^a^	0.98 ± 0.07 ^a^	0.95 ± 0.05 ^a^
Tyrosine	0.68 ± 0.06	0.68 ± 0.03	0.69 ± 0.11	0.61 ± 0.10
ΣΕAA	8.88 ± 0.68 ^a^	7.56 ± 0.53 ^b^	7.44 ± 0.65 ^b^	7.16 ± 0.71 ^b^
ΣΝΕAA	9.62 ± 0.82 ^a^	9.12 ± 0.94 ^b^	9.09 ± 0.91 ^b^	9.09 ± 0.80 ^b^
ΣAA	18.50 ± 1.16 ^a^	16.58 ± 1.03 ^b^	16.53 ± 1.11 ^b^	16.25 ± 1.18 ^b^
EAA/NEAA	0.92 ± 0.05 ^a^	0.83 ± 0.03 ^b^	0.82 ± 0.04 ^b^	0.79 ± 0.04 ^b^

^1^ In the experimental groups, CF: only commercial pellets; MTF: minced trash fish; MIX: pellets and minced trash fish mixed at a ratio of 50:50 were used as feed. ^a–d^ Mean ± SD followed by different letters within a row are significantly different (*p* < 0.05). Data are expressed as mean ± SD (*n* = 3).

## Data Availability

The original contributions presented in this study are included in the article. Further inquiries can be directed to the corresponding author.

## Abbreviations

The following abbreviations are used in this manuscript:

EAAs	Essential Amino Acids
NEAAs	Non-essential Amino Acids
GC	Gas chromatography
RAS	Recirculating aquaculture system
EU	European Union
DO	Dissolved Oxygen
